# Supplementary material for: Age, sex, and other demographic trends in sexual behavior in the United States: Initial findings of the sexual behaviors, internet use, and psychological adjustment survey
Source: PLoS One. 2021 Aug 6;16(8):e0255371. doi: 10.1371/journal.pone.0255371 (PMC8345845; doi:10.1371/journal.pone.0255371)
Supplement: S1 Table — (DOCX) [file pone.0255371.s001.docx]

**S1 Table. Effects of Age and Biological Sex on Prevalence Rates of Oral, Vaginal and Anal Sex.**

|  |  | Age | | |  |  |  | Age^2^ |  |  |  | Sex |  |  |  | Age x Sex | | |  | |  |  | Age^2^ x Sex | | |  |
| --- | --- | --- | --- | --- | --- | --- | --- | --- | --- | --- | --- | --- | --- | --- | --- | --- | --- | --- | --- | --- | --- | --- | --- | --- | --- | --- |
|  | b | | SE | OR | |  | b | SE | OR |  | b | SE | OR |  | b | | SE | OR | |  | | b | | SE | OR | |
| **Lifetime** |  | |  |  | |  |  |  |  |  |  |  |  |  |  | |  |  | |  | |  | |  |  | |
| Oral sex | .006 | | .005 | 1.01 | |  | **-.002** | .000 | 1.00 |  | .002 | .195 | 1.00 |  | .017 | | .007 | 1.02 | |  | | .001 | | .001 | 1.00 | |
| Vaginal sex | **.032** | | .007 | 1.03 | |  | **-.002** | .000 | 1.00 |  | -.561 | .232 | .57 |  | .003 | | .009 | 1.00 | |  | | .002 | | .001 | 1.00 | |
| Anal sex | **-.014** | | .005 | .99 | |  | **-.002** | .000 | 1.00 |  | -.112 | .141 | .89 |  | **.021** | | .007 | 1.02 | |  | | .000 | | .000 | 1.00 | |
| **Past Year** |  | |  |  | |  |  |  |  |  |  |  |  |  |  | |  |  | |  | |  | |  |  | |
| Oral sex | **-.049** | | .005 | .95 | |  | **-.002** | .000 | 1.00 |  | .105 | .145 | 1.11 |  | **.024** | | .007 | 1.02 | |  | | .001 | | .000 | 1.00 | |
| Vaginal sex | **-.035** | | .005 | .97 | |  | **-.002** | .000 | 1.00 |  | -.158 | .151 | .85 |  | **.018** | | .007 | 1.02 | |  | | **.001** | | .000 | 1.00 | |
| Anal sex | **-.057** | | .011 | .94 | |  | **-.002** | .001 | 1.00 |  | **.677** | .184 | 1.97 |  | .010 | | .013 | 1.01 | |  | | .000 | | .001 | 1.00 | |
| **Past 30 Days** |  | |  |  | |  |  |  |  |  |  |  |  |  |  | |  |  | |  | |  | |  |  | |
| Oral sex | **-.047** | | .006 | .95 | |  | **-.002** | .000 | 1.00 |  | .282 | .142 | 1.33 |  | **.020** | | .007 | 1.02 | |  | | .000 | | .000 | 1.00 | |
| Vaginal sex | **-.038** | | .005 | .96 | |  | **-.002** | .000 | 1.00 |  | -.168 | .143 | .85 |  | .018 | | .007 | 1.02 | |  | | **.001** | | .000 | 1.00 | |
| Anal sex | **-.047** | | .012 | .95 | |  | **-.002** | .001 | 1.00 |  | **.804** | .204 | 2.24 |  | .001 | | .014 | 1.00 | |  | | .000 | | .001 | 1.00 | |

***Note.*** b = unstandardized regression coefficients; SE = standard error; OR = odd ratio; Sex coded 0 = female, 1 = male; bold indicates *p* < .005.
