# Supplementary material for: Age, sex, and other demographic trends in sexual behavior in the United States: Initial findings of the sexual behaviors, internet use, and psychological adjustment survey
Source: PLoS One. 2021 Aug 6;16(8):e0255371. doi: 10.1371/journal.pone.0255371 (PMC8345845; doi:10.1371/journal.pone.0255371)
Supplement: S2 Table — (DOCX) [file pone.0255371.s002.docx]

**S2 Table. Effects of Age and Biological Sex on Frequency of Oral, Vaginal, and Anal Sex in the Past 12 months and Past 30 days (*N* = 1,987; Men *n* = 953, Women *n* = 1015)**

|  | Age | | |  | Age^2^ | | |  | Sex | | |  | Age x Sex | | |  | Age^2^ x Sex | | |
| --- | --- | --- | --- | --- | --- | --- | --- | --- | --- | --- | --- | --- | --- | --- | --- | --- | --- | --- | --- |
| Frequency | b | SE | β |  | b | SE | β |  | b | SE | β |  | b | SE | β |  | b | SE | β |
| **Past 12 months** |  |  |  |  |  |  |  |  |  |  |  |  |  |  |  |  |  |  |  |
| Oral sex | **-.037** | .004 | -.320 |  | **-.001** | .000 | -.170 |  | .218 | .122 | .059 |  | .007 | .005 | .042 |  | .000 | .000 | .035 |
| Vaginal sex | **-.042** | .004 | -.328 |  | **-.002** | .000 | -.222 |  | -.114 | .134 | -.028 |  | .014 | .006 | .080 |  | .001 | .000 | .087 |
| Anal sex | **-.011** | .002 | -.153 |  | .000 | .000 | -.033 |  | **.432** | .077 | .189 |  | -.007 | .003 | -.072 |  | .000 | .000 | -.080 |
| **Past 30 Days** |  |  |  |  |  |  |  |  |  |  |  |  |  |  |  |  |  |  |  |
| Oral sex | **-.034** | .004 | -.288 |  | **-.001** | .000 | -.151 |  | .280 | .125 | .074 |  | .005 | .005 | .031 |  | <.001 | .000 | .011 |
| Vaginal sex | **-.040** | .004 | -.309 |  | **-.002** | .000 | -.212 |  | -.080 | .137 | -.019 |  | .010 | .006 | .056 |  | .001 | .000 | .090 |
| Anal sex | **-.008** | .002 | -.118 |  | .000 | .000 | -.027 |  | **.422** | .074 | .193 |  | -.008 | .003 | -.082 |  | .000 | .000 | -.078 |

***Note.*** b = unstandardized regression coefficients; SE = standard error; β = standardized regression coefficients; Sex coded 0 = female, 1 = male; bold indicates *p* < .005.
