# Supplementary material for: Age, sex, and other demographic trends in sexual behavior in the United States: Initial findings of the sexual behaviors, internet use, and psychological adjustment survey
Source: PLoS One. 2021 Aug 6;16(8):e0255371. doi: 10.1371/journal.pone.0255371 (PMC8345845; doi:10.1371/journal.pone.0255371)
Supplement: S3 Table — (DOCX) [file pone.0255371.s003.docx]

**S3 Table. Effects of Age and Biological Sex on Number of Oral, Vaginal, and Anal Sex Partners for Lifetime and the Past 12 months (*N* = 1,987; Men *n* = 953, Women *n* = 1015).**

| Number of Partners | Age | | |  | Age^2^ | | |  | Sex | | |  | Age x Sex | | |  | Age^2^ x Sex | | |
| --- | --- | --- | --- | --- | --- | --- | --- | --- | --- | --- | --- | --- | --- | --- | --- | --- | --- | --- | --- |
|  | b | SE | β |  | b | SE | β |  | b | SE | β |  | b | SE | β |  | b | SE | β |
| **Lifetime** |  |  |  |  |  |  |  |  |  |  |  |  |  |  |  |  |  |  |  |
| Oral sex | -.059 | .033 | -.061 |  | **-.007** | .002 | -.104 |  | **5.837** | 1.061 | .188 |  | **.151** | .046 | .109 |  | -.003 | .003 | -.046 |
| Vaginal sex | -.035 | .039 | -.030 |  | **-.011** | .003 | -.135 |  | **4.745** | 1.264 | .128 |  | **.193** | .055 | .117 |  | .000 | .004 | .002 |
| Anal sex | -.019 | .014 | -.046 |  | -.002 | .001 | -.084 |  | **3.101** | .444 | .238 |  | -.002 | .019 | -.004 |  | -.003 | .001 | -.094 |
| **Past 12 months** |  |  |  |  |  |  |  |  |  |  |  |  |  |  |  |  |  |  |  |
| Oral sex | **-.024** | .005 | -.162 |  | .000 | .000 | -.017 |  | **.892** | .161 | .188 |  | -.009 | .007 | -.041 |  | -.001 | .000 | -.056 |
| Vaginal sex | **-.025** | .005 | -.169 |  | .000 | .000 | -.013 |  | **.768** | .160 | .162 |  | -.017 | .007 | -.079 |  | .000 | .000 | -.033 |
| Anal sex | -.010 | .005 | -.069 |  | .000 | .000 | -.018 |  | **.836** | .155 | .184 |  | **-.022** | .007 | -.110 |  | -.001 | .000 | -.055 |

***Note.*** b = unstandardized regression coefficients; SE = standard error; β = standardized regression coefficients; Sex coded 0 = female, 1 = male; bold indicates *p* < .005.
