# Supplementary material for: Age, sex, and other demographic trends in sexual behavior in the United States: Initial findings of the sexual behaviors, internet use, and psychological adjustment survey
Source: PLoS One. 2021 Aug 6;16(8):e0255371. doi: 10.1371/journal.pone.0255371 (PMC8345845; doi:10.1371/journal.pone.0255371)
Supplement: S4 Table — (DOCX) [file pone.0255371.s004.docx]

**S4 Table. Effects of Age and Biological Sex on Age of Initiation of Oral (Men *n* = 801, Women *n* = 819), Vaginal (Men *n* = 840, Women *n* = 879), and Anal Sex Partners (Men *n* = 372, Women *n* = 378).**

|  | Age | | |  | Age^2^ | | | |  | Sex | | | |  | Age x Sex | | |  | Age^2^ x Sex | | |
| --- | --- | --- | --- | --- | --- | --- | --- | --- | --- | --- | --- | --- | --- | --- | --- | --- | --- | --- | --- | --- | --- |
|  | b | SE | β |  | b | SE | | β |  | b | SE | | β |  | b | SE | β |  | b | SE | β |
| Oral sex | **.130** | .014 | .346 |  | .000 | .001 | .005 | |  | **-1.253** | | .426 | -.107 |  | -.039 | .019 | -.075 |  | .002 | .001 | .078 |
| Vaginal sex | **.055** | .011 | .182 |  | -.001 | .001 | -.053 | |  | -.549 | | .345 | -.058 |  | -.019 | .016 | -.045 |  | .002 | .001 | .076 |
| Anal sex | **.227** | .030 | .406 |  | **-.007** | .002 | -.169 | |  | **-4.117** | | .778 | -.254 |  | .015 | .041 | .020 |  | **.009** | .003 | .211 |

***Note.*** b = unstandardized regression coefficients; SE = standard error; β = standardized regression coefficients; Sex coded 0 = female, 1 = male; bold indicates *p* < .005.
